# Real world experience in low-dose ipilimumab in combination with PD-1 blockade in advanced melanoma patients

**DOI:** 10.18632/oncotarget.25627

**Published:** 2018-06-22

**Authors:** Michael Constantin Kirchberger, Alvaro Moreira, Michael Erdmann, Gerold Schuler, Lucie Heinzerling

**Affiliations:** ^1^ Department of Dermatology, University Hospital Erlangen, Friedrich-Alexander-University Erlangen-Nürnberg (FAU), Ulmenweg 18, 91054 Erlangen, Germany

**Keywords:** ipilimumab, pembrolizumab, nivolumab, low-dose, melanoma

## Abstract

Dual immune-checkpoint blockade with the anti-PD-1 antibody nivolumab (1 mg/kg) and standard-dose ipilimumab (3 mg/kg) is the mainstay of immunotherapy in advanced melanoma and it is approved since 2016. However, severe side effects (grade 3/4) occur in up to 60% of the patients. Recently, clinical trials have shown similar anti-tumor activity with a more favorable toxicity profile in patients treated with low-dose ipilimumab (1 mg/kg) and standard-dose pembrolizumab (2 mg/kg). In this study we report on the real-world experience of this dosing regime in advanced melanoma patients not eligible for clinical trials. A total of 33 patients with metastatic melanoma (24 with cutaneous and 9 with uveal melanoma) were assessed, retrospectively. Brain metastases were present in 33% of the patients and lactate dehydrogenase was elevated in 70%. Overall response rates were 38% and 0% in cutaneous melanoma and uveal melanoma respectively. Median overall survival was not reached in cutaneous melanoma and was 18 months in uveal melanoma. In 18% of the patients at least one treatment-related severe adverse event was observed. Our observation that the combination of standard dose pembrolizumab and low-dose ipilimumab has a favorable toxicity profile yet anti-tumor activity comparable to the approved standard-dose combination regime in advanced patients not suitable for enrollment in clinical trials is encouraging.

## INTRODUCTION

In recent years, monoclonal antibodies blocking the inhibitory programmed cell death 1 pathway (PD-1/PD-L1) and the cytotoxic T lymphocyte associated protein 4 (CTLA-4) have significantly impacted the treatment of advanced melanoma. The objective response rates for the anti-CTLA-4 antibody ipilimumab, the anti-PD-1 antibodies nivolumab and pembrolizumab, and the combined blockade of CTLA-4 and PD-1 are 6%-19%, 21%-44%, and 53%-61%, respectively [1–7]. In patients with brain metastases intracranial and overall response rate was 42% and 55% [8, 9]. However, the combination of CTLA-4 and PD-1 blockade also induces considerable toxicity with the highest frequency of grade 3/4 adverse events (AEs) (53%-59%) compared to anti-PD-1 (21%) or -CTLA-4 monotherapy (28%) [4, 10, 11].

Several clinical trials are modifying treatment protocols to optimize the cost-benefit ratio of response rate, survival and toxicity of checkpoint inhibitors. In metastatic renal cell carcinoma efficacy as assessed by ORR and progression-free survival (PFS) did not differ between ipilimumab 3 mg/kg + nivolumab 1 mg/kg and ipilimumab 1 mg/kg + nivolumab 3 mg/kg, however, the low-dose ipilimumab group showed a better safety profile [12]. In melanoma the low-dose ipilimumab (1 mg/kg) protocol was investigated in the KEYNOTE-029 (NCT02089685) and CheckMate 511 (NCT02714218) trials. Long et al. published results (KEYNOTE-029) of 153 patients showing an ORR of 61% and a disease control rate (DCR) of 79% with 45% grade 3/4 treatment-related [13]. Reducing the toxicity by the sequential administration of Ipilimumab (3 mg/kg, four doses), followed by nivolumab (3 mg/kg) versus vice versa has also been investigated (Checkmate 064 trial) [14]. Notably, the rate of grade 3/4 AEs between both cohorts was also high with 63% in the nivolumab followed by ipilimumab group and 50% in the reverse sequence. Reducing the number of ipilimumab infusions was investigated in a cohort of 40 patients with short-term ipilimumab (2 cycles) followed by PD-1 blockade and induced treatment-related grade 3/4 AEs in only 38% of patients with an ORR of 55% [15]. Thus, sequencing and dosing of anti-CTLA-4 and anti-PD-1 plays a significant role regarding efficacy and toxicity. However, the best regime is still to be determined.

This study analyzes the clinical course of 33 patients with advanced metastatic melanoma who received the combination of low-dose ipilimumab (1 mg/kg) and standard-dose anti-PD-1 antibodies (2mg/kg) in a real-world setting. We report clinical outcomes with respect to response, survival and tolerability.

## RESULTS

**Figure 1:**
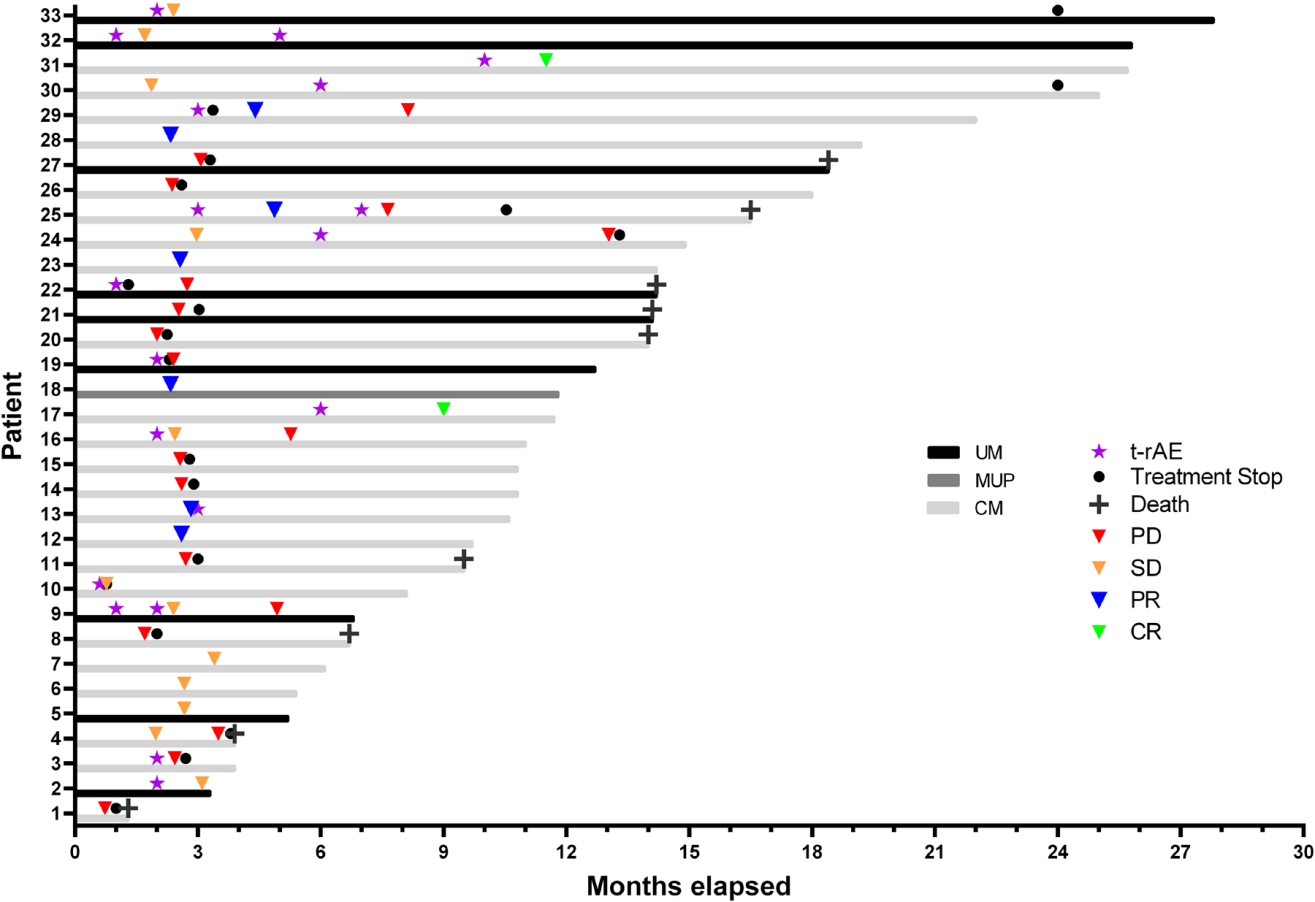
Swimmers plot of melanoma patients treated with low-dose ipilimumab and standard-dose pembrolizumab UM = uveal melanoma (N = 9), MUP = melanoma of unknown primary (N = 1), CM = cutaneous melanoma (N = 23), t-rAE = treatment-related adverse event, PD = progressive disease, SD = stable disease, PR = partial response, CR = complete response.

A total of 33 patients were included in this study (Figure 1), 23 patients with cutaneous melanoma, 9 patients with uveal melanoma and 1 patient with melanoma of unknown primary (MUP; Table 1). The patient with MUP harbored a v-Raf murine sarcoma viral oncogene homolog (BRAF) V600E mutation and was assigned to the group of cutaneous melanomas. Median follow-up was 12 months with a cutoff date as of September 2017.

**Table 1:** Baseline characteristics of the patients

Characteristic	Cutaneous Melanoma	Uveal Melanoma
**Age at stage IV (years)**		
Median (range)	57 (32-87)	66 (54-75)
**Sex, N (%)**		
Female	6 (24)	3 (33)
Male	18 (75)	6 (67)
**ECOG performance status**		
0	15 (63)	4 (45)
1	7 (29)	3 (33)
2	2 (8)	2 (22)
**Metastatic site, N (%)**		
Brain	10 (42)	1 (11)
Liver	8 (33)	8 (88)
Lung	11 (46)	3 (33)
Nodal/cutaneous	18 (75)	3 (9)
Bone	6 (25)	0 (0)
Other	6 (25)	1 (11)
**Mutation status, N (%)**		
NRAS/BRAF wildtype	10 (42)	NA
BRAFV600	12 (50)	NA
NRAS	2 (8)	NA
**Prior treatment, N (%)**		
None	20 (83)	1 (11)
BRAF- and MEK-Inhibitors	4 (17)	0 (0)
Chemotherapy	1 (4)	0 (0)
Liver-specific therapies	0 (0)	8 (89)
**Lactate dehydrogenase**		
Median (range)	339 (160-2353)	278 (216-407)
≤ ULN (N, (%))	6 (25)	4 (44)
> ULN	18 (75)	5 (56)
**S100B**		
Median (range)	0.24 (0.03-22.65)	0.06
≤ ULN (N, (%))	6 (25)	8 (89)
> ULN	18 (75)	1 (11)
**MIA**		
Median (range)	23.1 (2.9-161.2)	10.1 (4.3-30.4)
≤ ULN (N, (%))	7 (33)	4 (50)
> ULN	14 (67)	4 (50)
**CRP**		
Median (range)	5.8 (3.3-105.3)	1.5 (1.1-11.9)
≤ ULN (N, (%))	7 (41)	2 (67)
> ULN	10 (59)	1 (33)
**REC (%)**		
Median (range)	2 (0.1-5.0)	1 (0.1-7.0)

Abbreviations: ECOG = Eastern Cooperative Oncology Group, BRAF = v-Raf murine sarcoma viral oncogene homolog, NRAS = neuroblastoma rat sarcoma viral oncogene homolog, S100B = S100 calcium-binding protein B, MIA = melanoma inhibitory activity, CRP = c-reactive protein, REC = relative eosinophil counts, ULN = upper limit of normal.

In 17% (*N* = 4) of the patients with cutaneous melanoma, prior treatment was recorded and consisted of targeted therapy with BRAF and MEK inhibitors (33% of BRAF mutant patients; *N* = 4). One patient had radiochemotherapy prior to targeted therapy. Treatment stop of prior treatment was due to progressive disease in all of the cases.

In 89% (*N* = 8) of the patients with uveal melanoma, hepatic metastases were present and prior treatment with either selective internal radiotherapy (*N* = 4), radiofrequency ablation (*N* = 1) or microwave ablation (*N* = 1) was performed. In two cases, these therapeutic modalities were omitted due to the extent of metastases.

**Figure 2:**
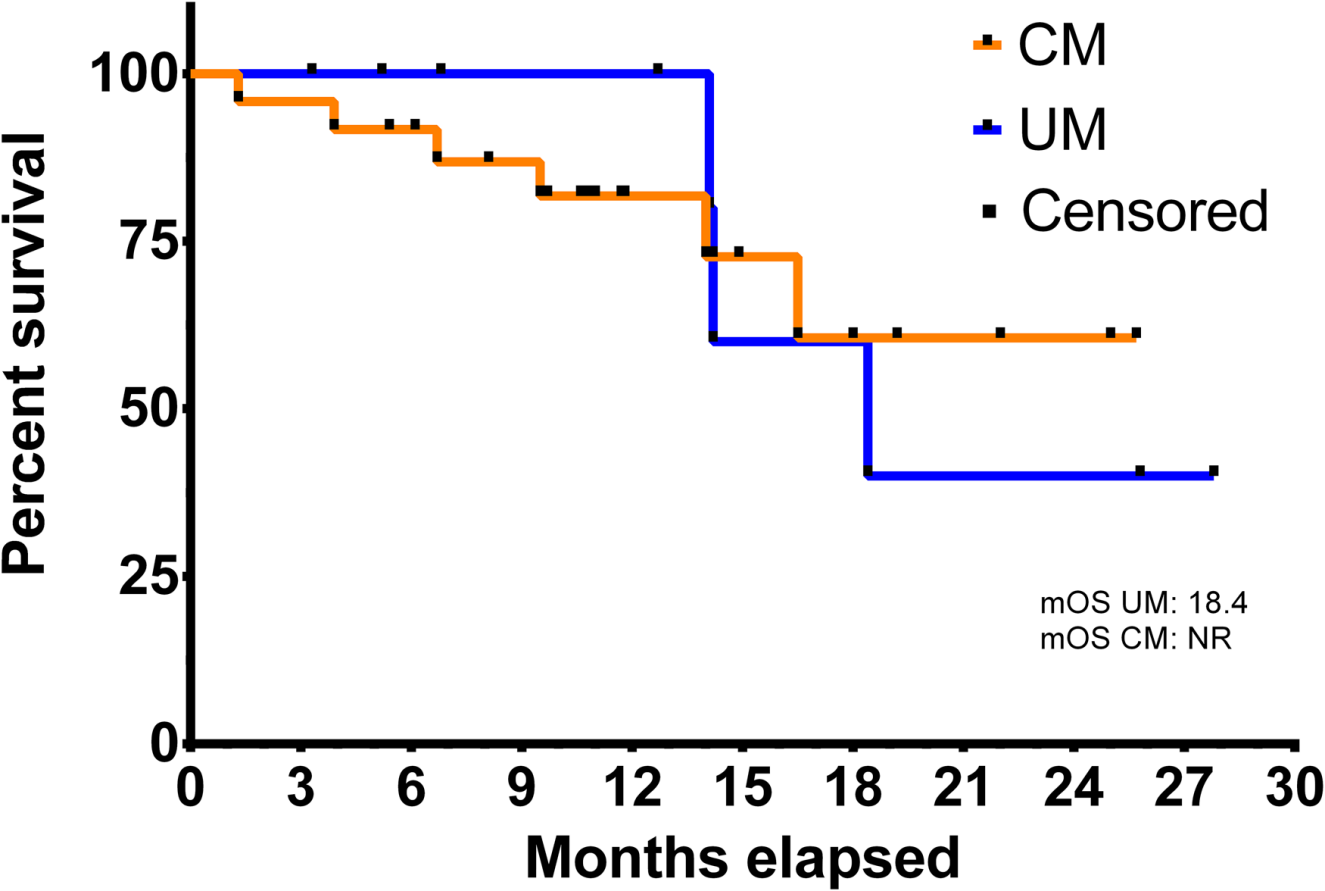
Overall survival from initiation of checkpoint inhibitor therapy in uveal (UM, N = 9) and cutaneous melanoma (CM, N = 24) mOS = median overall survival, NR = not reached.

First line immunotherapy was combined pembrolizumab (2 mg/kg) and low-dose ipilimumab (1 mg/kg) for 4 infusions every three weeks in all patients. In cutaneous melanoma (*N* = 24) ORR was 38% (*N* = 9) and DCR 67% (*N* = 16; Table 2). Patients with uveal melanoma had an ORR of 0% (*N* = 0) and a DCR of 56% (*N* = 5). Median time to best response and treatment stop was 3 months. Monotherapy with pembrolizumab (2 mg/kg) was continued after combined immunotherapy in 46% (*N* = 13) of the patients with cutaneous melanoma and 44% (*N* = 4) of the patients with uveal melanoma. The median overall survival (OS) from initiation of ipilimumab and pembrolizumab was 18.4 months in the uveal melanoma group and was not reached for cutaneous melanoma (Figure 2; *P* = 0.005). The most common reasons for discontinuation of treatment were disease progression (42%; *N* = 14) and adverse events (AEs; 18%; *N* = 6). Two patients (6%) discontinued therapy after 24 months and achieved SD during the treatment period.

**Table 2:** Treatment response to combined immunotherapy with ipilimumab and pembrolizumab in cutaneous and uveal melanoma

Best Response, N (%)	Cutaneous Melanoma (N = 24)	Uveal Melanoma (N = 9)
PD	8 (33)	4 (44)
SD	7 (29)	5 (56)
PR	7 (29)	0 (0)
CR	2 (8)	0 (0)

Abbreviations: PD = progressive disease, SD = stable disease, PR = partial response, CR = complete response.

**Figure 3:**
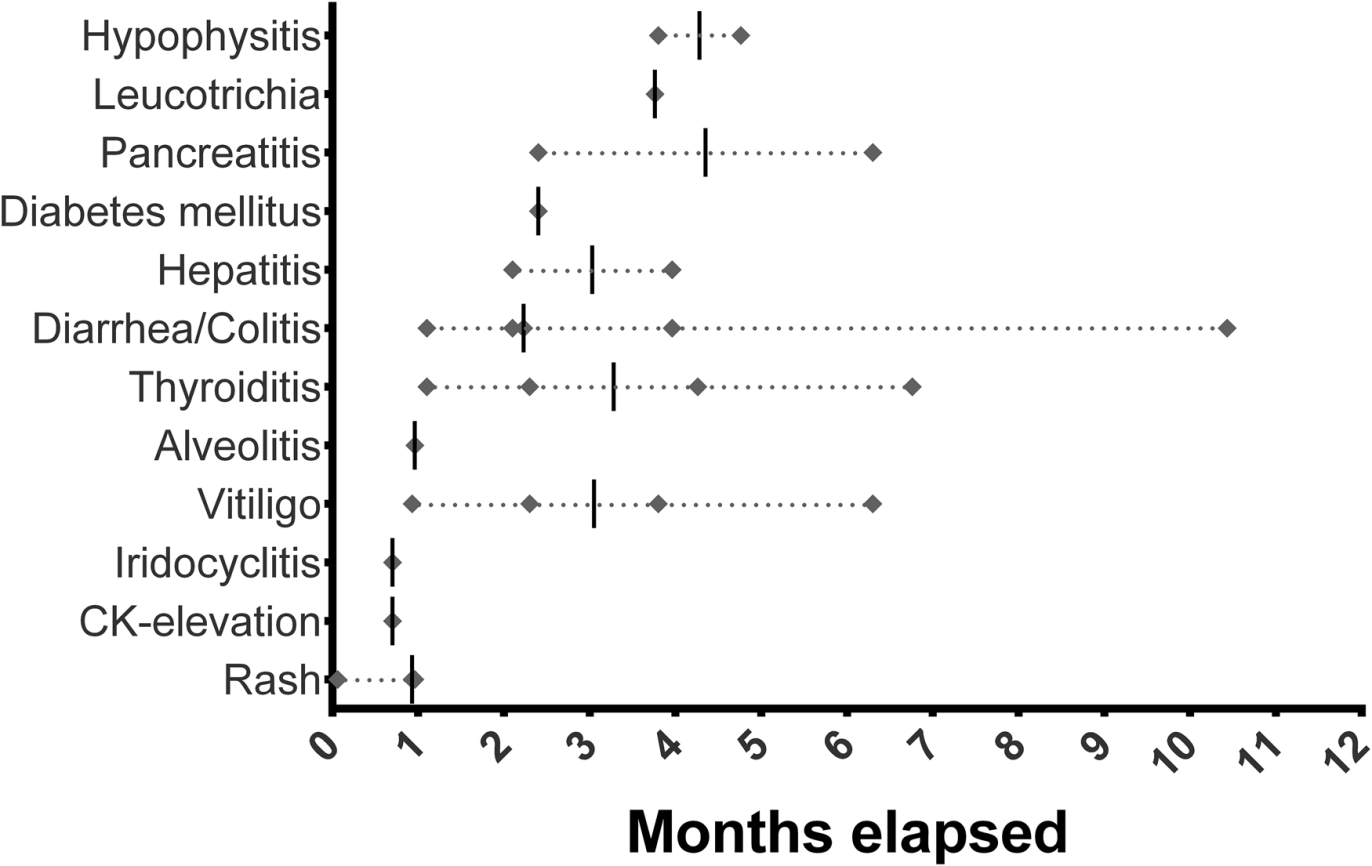
Temporal occurrence of treatment-related adverse events (Diamond) in months Bar = median, CK = creatine kinase.

In 61% (*N* = 20) of the patients at least one treatment-related AE was recorded, including 18% (*N* = 6) who had at least of AE of grade 3 or 4 severity. No treatment-related deaths occurred. Most common treatment-related AEs comprised colitis, diarrhea, thyroiditis and vitiligo with a median time of onset after 10 weeks (Figure 3). In 10 patients, treatment-related AEs occurred before response (SD, PR or CR) to therapy with a median time of onset of 4 weeks after the AE.

## DISCUSSION

In this study we show that combined standard-dose pembrolizumab (2 mg/kg) with low-dose ipilimumab (1 mg/kg) has a strikingly better toxicity profile yet similar anti-tumor activity as standard combination therapy. ORR was 38% in this patient cohort with unfavorable prognostic markers (33% brain metastases, 70% elevated LDH) compared to 58% and grade 3/4 side effects occurred in only 18% compared to 59% [11]. However, in this real-world setting, registration of AEs is somewhat less than in clinical trials. Reducing severe treatment-related AEs remains a main concern and multiple studies are evaluating whether or not reduction of the dose or frequency of administration can improve tolerability at similar efficacy [13, 16–18]. Interestingly, our rate of grade 3/4 side effects with 18% was even lower than the 45% in the Keynote-029 trial [13]. However, this difference could be attributed to the small cohort and the less rigorous surveillance of AEs in a real-world setting. Also differences between subgroups might have been missed due to the limited cohort size.

In terms of disease control, our cohort showed a DCR of 67% in cutaneous melanoma compared to 79% in the Keynote-029 trial [13]. Baseline characteristics may account for that difference since patients in our cohort were not suitable for participation in clinical trials. In cutaneous and uveal melanoma elevated LDH was observed in 75% and 56%, respectively. Also brain metastasis was present in 44% of the cutaneous melanoma and in 11% of the uveal melanoma.

Interestingly, in 10 patients treatment-related AEs were observed before, and in 1 patient shortly after positive response to immunotherapy, compared to 5 patients who suffered PD after treatment-related AEs. Vitiligo as treatment-related AE is typically observed in melanoma patients treated with anti-PD-1 antibodies [19] and indicated positive response to therapy in 4 of our patients (Figure 1, Patient 2, 9, 25, 30). However, the predictive use of treatment-related AEs to therapy response has to be further investigated.

## MATERIALS AND METHODS

This retrospective analysis included checkpoint-blockade naïve advanced cutaneous and uveal melanoma patients treated with combined immune checkpoint inhibitors ipilimumab (1 mg/kg, q3w in combination) and pembrolizumab (2 mg/kg, subsequently q3w) at the skin cancer center Erlangen between January 2016 and September 2017. Patients were screened using pharmacy records and clinical data and treatment outcomes were extracted from pre-existing patient records in the eligible cases.

Clinical data taken at baseline before initiation of the therapy comprised demographics with Eastern Cooperative Oncology Group (ECOG) performance status, mutation status, metastatic sites, and prior systemic antineoplastic therapies. In addition, laboratory parameters such as lactate dehydrogenase (LDH), C-reactive protein (CRP), eosinophils (REC), S100B and melanoma inhibitory activity (MIA) were gathered and analyzed.

Tumor response was assessed every 3 months by CT- and MRI-scans by a radiologist according to the Response Evaluation Criteria in Solid Rumors (RECIST) version 1.1. to determine DCR (CR rate plus PR rate plus rate of SD) and ORR (CR rate plus PR rate). However, if progressive disease was suspected clinically or due to increasing tumor markers, imaging was performed immediately. Overall survival and PFS were calculated as time from the initiation of the therapy until melanoma-specific or treatment-related death and disease progression, respectively. They were computed with time-to-event analyses with death and progression considered as events. If no such event occurred or if patients were lost to follow-up, the date of the last documented contact was registered and used as censored observation. The survival and progression probabilities were calculated with the Kaplan-Meier method for censored failure time data assuming proportional hazards.

Toxicity was graded based on the patient records and clinical outcomes according to the Common Terminology Criteria for Adverse Events (CTCAE) v4.03 published by the National Institutes of Health in 2010. In addition, treatment-related deaths and treatment discontinuation due to severe AE were specifically collected. Patients completed a questionnaire to screen for AEs at the time of each infusion. A physician controlled the questionnaire and performed a physical examination.

This study was approved by the institutional review board of the medical faculty of the University Erlangen and was conducted in accordance with the principles of the Helsinki Declaration of 1975.
